# A Bacterial Cytotoxin Identifies the RhoA Exchange Factor Net1 as a Key Effector in the Response to DNA Damage

**DOI:** 10.1371/journal.pone.0002254

**Published:** 2008-05-28

**Authors:** Lina Guerra, Heather S. Carr, Agneta Richter-Dahlfors, Maria G. Masucci, Monica Thelestam, Jeffrey A. Frost, Teresa Frisan

**Affiliations:** 1 Department of Cell and Molecular Biology, Karolinska Institutet, Stockholm, Sweden; 2 Department of Neuroscience, Karolinska Institutet, Stockholm, Sweden; 3 Department of Integrative Biology and Pharmacology, University of Texas Health Science Center at Houston, Houston, Texas, United States of America; University of Birmingham, United Kingdom

## Abstract

**Background:**

Exposure of adherent cells to DNA damaging agents, such as the bacterial cytolethal distending toxin (CDT) or ionizing radiations (IR), activates the small GTPase RhoA, which promotes the formation of actin stress fibers and delays cell death. The signalling intermediates that regulate RhoA activation and promote cell survival are unknown.

**Principal Findings:**

We demonstrate that the nuclear RhoA-specific Guanine nucleotide Exchange Factor (GEF) Net1 becomes dephosphorylated at a critical inhibitory site in cells exposed to CDT or IR. Expression of a dominant negative Net1 or Net1 knock down by iRNA prevented RhoA activation, inhibited the formation of stress fibers, and enhanced cell death, indicating that Net1 activation is required for this RhoA-mediated responses to genotoxic stress. The Net1 and RhoA-dependent signals involved activation of the Mitogen-Activated Protein Kinase p38 and its downstream target MAPK-activated protein kinase 2.

**Significance:**

Our data highlight the importance of Net1 in controlling RhoA and p38 MAPK mediated cell survival in cells exposed to DNA damaging agents and illustrate a molecular pathway whereby chronic exposure to a bacterial toxin may promote genomic instability.

## Introduction

Cytolethal distending toxins (CDTs), produced by several pathogenic Gram-negative bacteria, are protein toxins which cause DNA damage (reviewed in [1]). The active holotoxin is a tripartite complex 2, 3, formed by the CdtA, CdtB and CdtC subunits, (reviewed in [1]). Cellular intoxication with CDT induces DNA double strand breaks and activation of checkpoint responses that, depending on the cell type, lead to arrest in the G1 or G2 phases of the cell cycle [4]–[6]. These effects are similar to those caused by ionizing radiation (IR), which is a well-characterized DNA-damaging agent. Activation of the DNA damage responses by CDT is consistent with the functional and structural homology of the CdtB subunit with mammalian DNase I [7]–[9].

In adherent cells, CDT intoxication and exposure to IR are associated with the formation of actin stress fibers, via activation of the small GTPase RhoA [6]. While a large amount of data is available regarding the activation of RhoA upon stimulation of plasma membrane-bound receptors [10], the molecular mechanisms regulating RhoA activation in response to these and other DNA-damaging agents are still unknown. It is noteworthy that, since RhoA activation occurs in the cytosol, the signals that regulate its activation in response to DNA damage must be then transduced from the nucleus.

Guanine nucleotide exchange factors (GEFs) are key activators of the small GTPases that regulate the switch between the inactive GDP-bound and the active GTP-bound forms of the GTPase (reviewed in [11]). The vast majority of the known RhoA-specific GEFs exhibit a cytoplasmic localization. One remarkable exception is the RhoA-specific GEF encoded by the neuroepithelioma transforming gene 1 (*Net1*) that is normally found in the nucleus of mammalian cells (reviewed in [11]). Net1 was originally isolated in a tissue culture screen for transforming genes in NIH 3T3 cell focus formation assays [12]. The oncogenic form of Net1 isolated from this screen lacked the first 145 amino acids. A deletion mutant of Net1 lacking the first 121 amino acids was shown to be constitutively active and induced: i) formation of actin stress fibers; ii) activation of the Mitogen-Activated Protein Kinase (MAPK) JNK; and iii) activation of the serum response factor (SRF) [13], [14].

The regulation of Net1 activity is poorly understood. PAK1-dependent phosphorylation of Net1 on Ser152 and Ser153 inhibits its GEF activity and abolishes Net1-dependent RhoA activation and stress fiber induction [15]. In addition, translocation of Net1 from the nucleus to the cytoplasm is required for activation of RhoA. The amino-terminus of Net1 contains multiple nuclear localization signals, and deletion of this domain is associated with accumulation of a constitutively active Net1 in the cytoplasm [14]. Taken together these data suggest that the activation of Net1 requires both changes in the phosphorylation pattern of specific inhibitory sites and shuttling between the nucleus and the cytoplasm, but the signals that trigger these events are still unknown.

In the present study, we have identified DNA damage as a trigger for Net1 activation. We show that inhibition of Net1 prevents RhoA activation and stress fiber formation, and promotes cell death upon intoxication or irradiation. We also demonstrate that the Net1/RhoA dependent signals converges on the activation of p38 MAPK and its downstream target MK2, indicating that RhoA plays an important role in controlling the activation of this MAPK pathway in response to genotoxic agents.

## Results

### DNA damage induces Net1 activation

We have previously shown that exposure to DNA damaging agents induces activation of the small GTPase RhoA, which delays cell death [6]. In order to investigate how the signal delivered by DNA damage is transduced from the nucleus to the cytosol, we have studied the activation of the RhoA specific GEF Net1 in response to CDT or IR. These genotoxic agents were chosen since they both induce DNA double strand breaks and activate identical DNA damage checkpoint responses in mammalian cells [4]–[6]. Decreased phosphorylation of Net1 on the key negative regulatory site Ser152 was used as the hallmark of Net1 activation [15].

**Figure 1:**
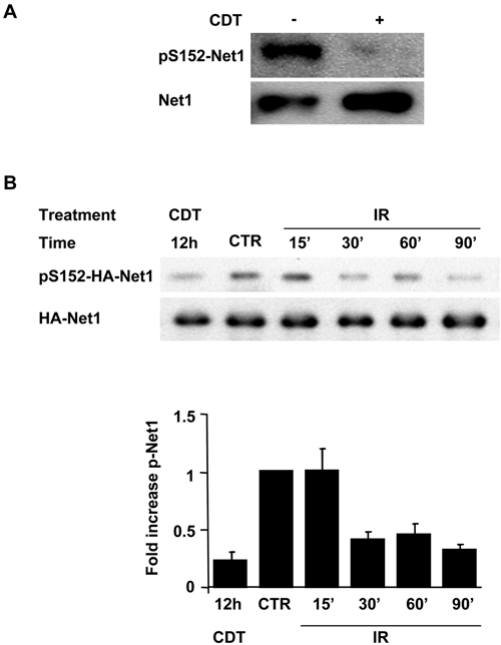
Decreased phosphorylation of Net1 upon induction of DNA damage. A) HeLa cells were left untreated or treated with CDT (2 μg ml^−1^) for 12h. The endogenous Net1 protein was immunoprecipitated using a goat α-Net1 antibody, and samples were analyzed by western blot using a rabbit serum specific for Net1 phosphorylated on Ser152 (pS152-Net1). B) HeLa cells, grown in 12-well plate, were transfected with a HA-epitope tagged Net1A expression plasmid (4 μg/well). Twenty-four hours after transfection, the cells were left untreated (indicated as CTR), or: i) irradiated (20 Gy), and incubated for the indicated time; ii) exposed to CDT (2 μg ml^−1^) for 12h. The HA-Net1A protein was immunoprecipitated using an α-HA antibody, and the levels of Net1 phosphorylated on Ser152 (pS152-HA-Net1) were assessed as in Figure 1A. The same membrane was re-probed with an α-HA antibody (ΗΑ-Net1). The fold increase represents the ratio between the levels of pS152-Net1 in treated cells and the levels of pS152-Net1 in untreated cells (mean±SD of three independent experiments).

Endogenous Net1 was immunoprecipitated from HeLa cells left untreated or exposed to CDT for 12h, and the levels of Ser152 phosphorylation were assessed by western blot using a phospho-specific antibody. As shown in Figure 1A, the phosphorylation of endogenous Net1 on Ser152 (pS152-Net1) was significantly decreased in intoxicated cells. To assess whether dephosphorylation of Net1 is a reproducible effect of DNA damage, HeLa cells were transfected with a plasmid expressing an HA-epitope-tagged Net1A, the major Net1 isoform expressed in these cells [16]. The transfected cells were then exposed to CDT or IR and the expression of total and phosphorylated Net1 was monitored over time. A 70% decrease in the levels of pS152-Net1A was observed within 30 min after irradiation. The effect was similar to that achieved by CDT intoxication where low levels of p152-Net1A were maintained for at least 12h (Figure 1B and data not shown). Thus, exposure to DNA damage induces dephosphorylation of Net1 on its negative regulatory site.

### Net1 is required for activation of RhoA and remodelling of the actin cytoskeleton in response to DNA damage

**Figure 2:**
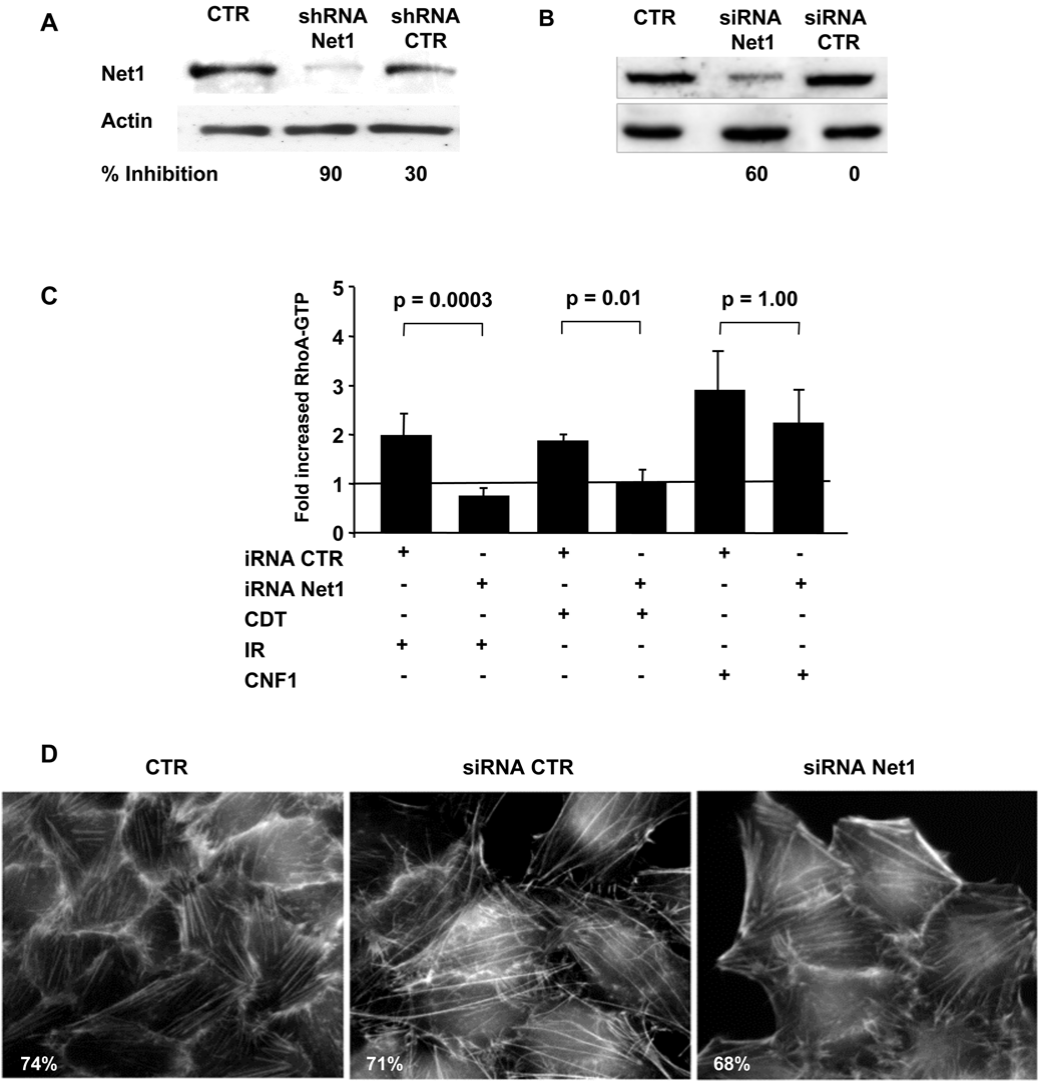
Net1 knock down prevents RhoA activation upon induction of DNA damage. HeLa cells were transfected with plasmids expressing the control or Net1 specific shRNAs (panel A), or control or Net1 specific siRNA (panel B). Expression of the endogenous Net1 was analysed by western blot 96h (A) or 72h (B) after transfection. Percentage inhibition was calculated as (1- residual Net1)×100, where residual Net1 is defined as the ratio between the optical density of the Net1 specific band in cells transfected with the Net1 iRNA or control iRNA and the optical density of the Net1 specific band in the non-transfected cells. C) HeLa cells, transfected with control or Net1 specific shRNA or shRNA, were left untreated, or exposed to IR (20 Gy), CDT (2 μg ml^−1^), or CNF-1 (1 μg ml^−1^), respectively, and further incubated for 4h. Activation of RhoA was assessed by RhoA specific G-LISA™ (mean ±SD of 5 independent experiments for IR and CNF, mean±SD of 3 independent experiments for CDT). Since the effects of transfection with specific Net1 shRNA or siRNA were similar, the data from all these experiments have been summarized together and indicated as iRNA. The fold increase represents the ratio between the levels of GTP-bound RhoA in treated cells and the levels of GTP-bound RhoA in untreated cells. According to the paired *t* test, the reduced RhoA activation in irradiated or intoxicated cells transfected with Net1 iRNA is statistically significant, while the effect of iRNA on the CNF1-induced RhoA activation is not statistically significant. D) HeLa cells non-transfected or transfected with control or Net1 specific siRNA were exposed to CNF1 (1 μg ml^−1^) for 6h. The actin cytoskeleton was visualized by TRITC-phalloidin staining. The values represent the percentage of cells with stress fibers. Cells exhibiting more than 5 stress fibers were scored as positive.

To assess whether Net1 is required for activation of RhoA, the expression of endogenous Net1 was knocked down by RNAi prior to intoxication or irradiation. Transfection with a short hairpin loop interfering RNA expressing plasmid or siRNA oligonucleotides were used to inhibit Net1 expression. Transfection of cells with Net1 specific shRNA resulted in 90% reduction of the endogenous Net1 96h after transfection (Figure 2A), while transfection with a specific siRNA resulted in 60 to 70 percent reduction 72h after transfection (Figure 2B). Since the effects of Net1 specific shRNA or siRNA were reproducibly similar, the results of Net1 knock down experiments have been summarized together and are henceforth indicated as iRNA. Consistent with our previous results [6], a 2- to 4-fold increase in the levels of GTP-bound RhoA was observed in irradiated or intoxicated HeLa cells as compared to the untreated controls. Knock down of endogenous Net1 blocked the activation of RhoA in response to IR or CDT (Figure 2C). Similar results were obtained in cells expressing the dominant negative Net1ΔDH (data not shown). This effect was not due to a general impairment of RhoA-dependent responses since Net1 knock down did not prevent RhoA activation or the formation of actin stress fibers in cells treated with cytotoxic necrotizing factor 1 (CNF1) that constitutively activates RhoA by deamidating Gln-63 and preventing hydrolysis of bound GTP (Figures 2C and 2D).

**Figure 3:**
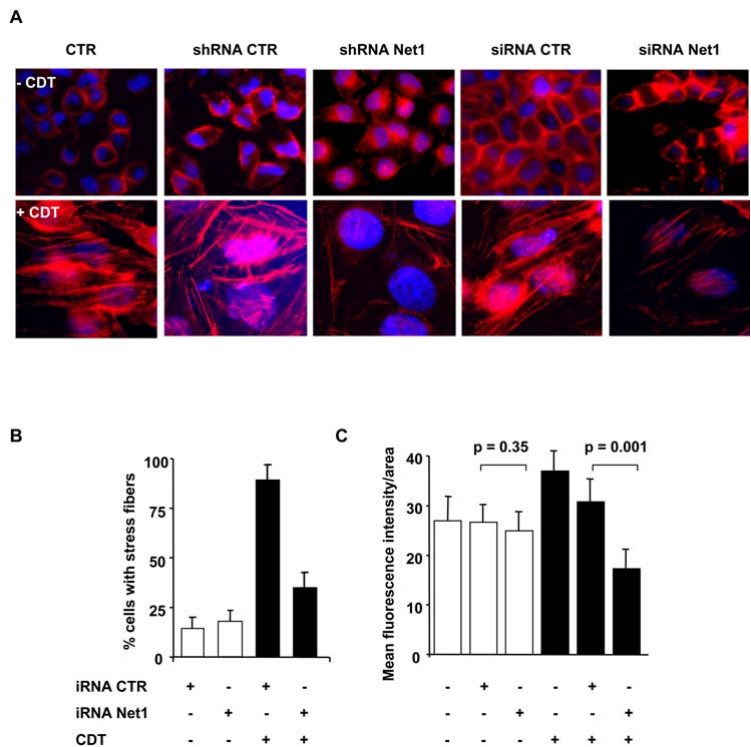
Net1 knock down prevents actin stress fiber formation upon intoxication. A) HeLa cells non-transfected, or transfected with control or Net1 specific shRNA or siRNA, were left untreated or treated with CDT (2 μg ml^−1^) for 24h. The actin cytoskeleton was visualized by TRITC-phalloidin staining (red). B) Quantification of cells with actin stress fibers (mean±SD of 5 independent experiments: three performed with shRNA and two performed with siRNA). One hundred and fifty cells were counted for each experiment. Cells carrying more than 5 stress fibers were scored as positive. C) The fluorescence intensity per each cell was quantified using the *ImageJ* software. Data are presented as ratio between the mean fluorescence intensity and the cell area. iRNA is defined as in Figure 2B.

We next examined whether blockade of Net1 affected the RhoA-dependent formation of actin stress fibers in HeLa cells exposed to CDT. Induction of stress fibers was detected upon intoxication in approximately 80% of control non-transfected cells or cells transfected with non-silencing siRNA or shRNA. In contrast, knock down of endogenous Net1 expression prior to intoxication resulted in significant reduction in the number of cells presenting actin stress fibers (Figures 3A and 3B). This effect was quantified by measuring the intensity of the phalloidin staining using the ImageJ software. A significant decrease in the intensity of fluorescence induced by intoxication was demonstrated in the Net1 RNAi treated cells as compared to controls (Figure 3C).

### Net1 regulates cell survival in response to DNA damage

**Figure 4:**
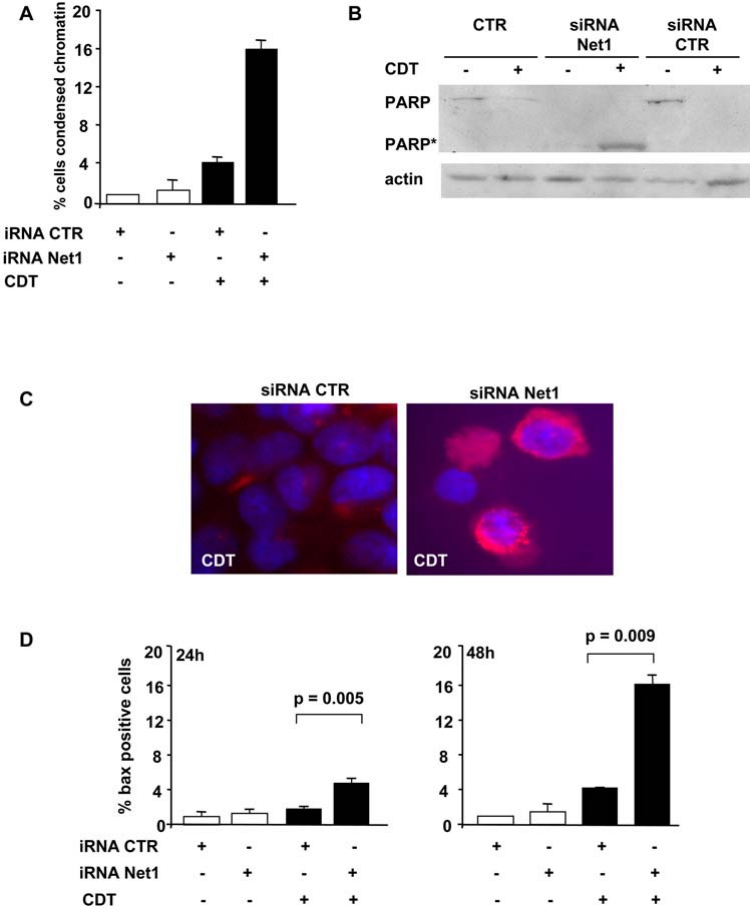
Net1 knock down increases the rate of cell death upon induction of DNA damage. HeLa cells were transfected with control or Net1 specific shRNA or siRNA and exposed to CDT (2 μg ml^−1^) for 48h. Cell death was assessed by quantifying the percentage of cells presenting chromatin condensation by Hoechst 33258 staining (panel A), detection of the cleaved form of PARP (PARP*) by western-blot (panel B) and detection of activated Bax, using the anti-Bax 6A7 antibody (red; panel C). D) Quantification of the Bax positive cells (mean±SD of 5 independent experiments: three performed with siRNA and two performed with shRNA). iRNA is defined as in Figure 2B. According to the *t* test, the increased number of cells expressing the activated form of Bax upon transfection with Net1 specific iRNA is statistically significant both at 24h and 48h after intoxication.

Activation of RhoA promotes the survival of cells exposed to CDT [6]. In order to investigate whether Net1 is required for this RhoA mediated response, Net1 expression was inhibited by RNAi prior to intoxication and cell death was assessed by monitoring chromatin condensation. Down-regulation of Net1 resulted in a 4- to 5-fold increase in the number of cells presenting chromatin condensation 48h after intoxication (Figure 4A). Similar results were obtained in cells expressing the dominant negative Net1ΔDH (data not shown). Induction of cell death upon Net1 knock down was confirmed by cleavage of the caspase-3 substrate PARP, as detected by western-blot analysis (Figure 4B), and activation of the pro-apoptotic protein Bax, as detected by immunostaining using the conformation-dependent antibody 6A7 (Figures 4C and 4D). The effect was already observed after 24h and became highly significant within 48h of treatment. The late occurrence of CDT-induced cell death observed upon Net1 knock down was similar to that observed in cells expressing a dominant negative RhoA (RhoAN19) [6]. These results indicate that Net1 is an essential component in the survival response to DNA damage.

### Net1/RhoA-dependent activation of p38 MAPK is required for cell survival in intoxicated or irradiated cells

**Figure 5:**
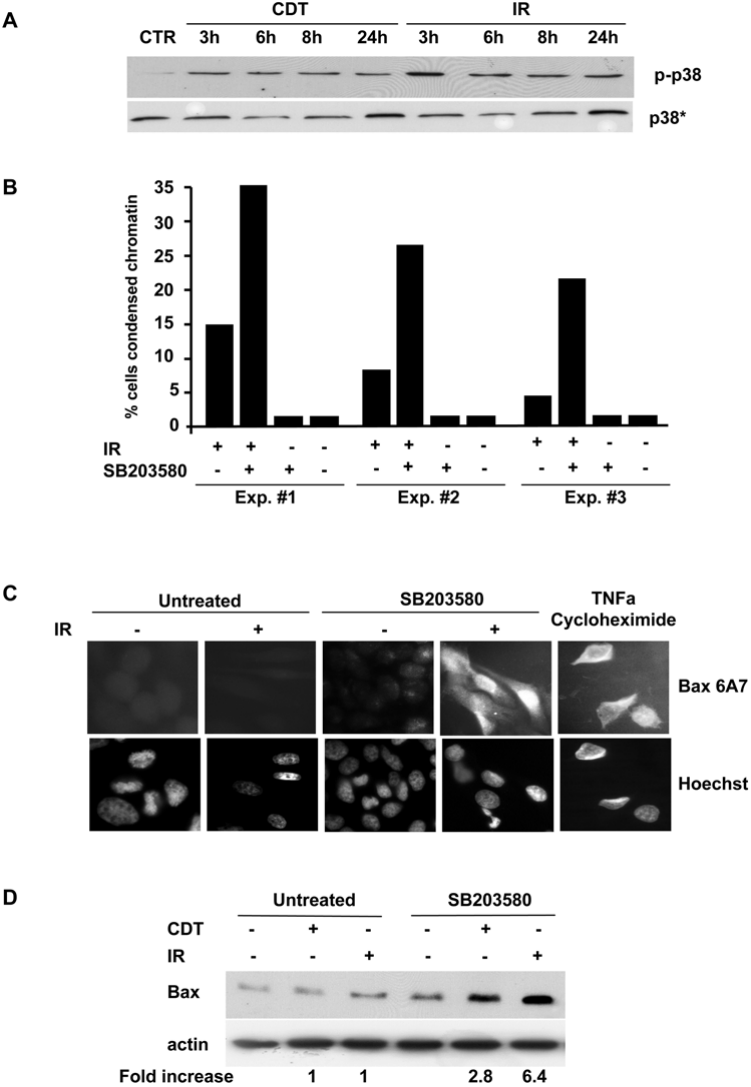
p38 MAPK regulates cell survival in response to DNA damage. A) HeLa cells were: i) left untreated; ii) exposed to CDT (2 μg ml^−1^) for the indicated time periods; iii) irradiated (20 Gy) and further incubated in complete medium for the indicated time periods. Samples were subjected to western blot analysis using a α-p38 antibody (p38*) or a p38 phospho-specific antibody (p-p38). B) HeLa cells, pre-treated with the specific p38 MAPK inhibitor SB203580 (20 μM) in complete medium for 30 min, were left untreated, or irradiated (20 Gy) and further incubated for 48h. Cell death was assessed by quantifying the number of cells presenting chromatin condensation by Hoechst 33258 staining. C) HeLa cells were treated as described in Figure 5B, and the activated form of Bax was detected by indirect immunofluorescence using the anti-Bax 6A7 antibody (upper panel). Nuclei were counterstained with Hoechst 33258 (lower panel). As a positive control for Bax staining the cells were treated with 50 ng/ml TNFα and 100 μg/ml cyclohexmide in complete medium for 6h at 37°C. D) HeLa cells, pre-treated with the specific p38 MAPK inhibitor SB203580 (20 μM) in complete medium for 30 min, were left untreated, treated with CDT (2 μg ml^−1^) for 48h, or irradiated (20 Gy) and further incubated for 48h. Expression of Bax was detected by western blot analysis using antibodies specific for Bax or actin. Fold increase represents the ratio between the optical density of the Bax specific band in treated cells and optical density of the Bax specific band in the untreated cells.

**Figure 6 pone-0002254-g006:**
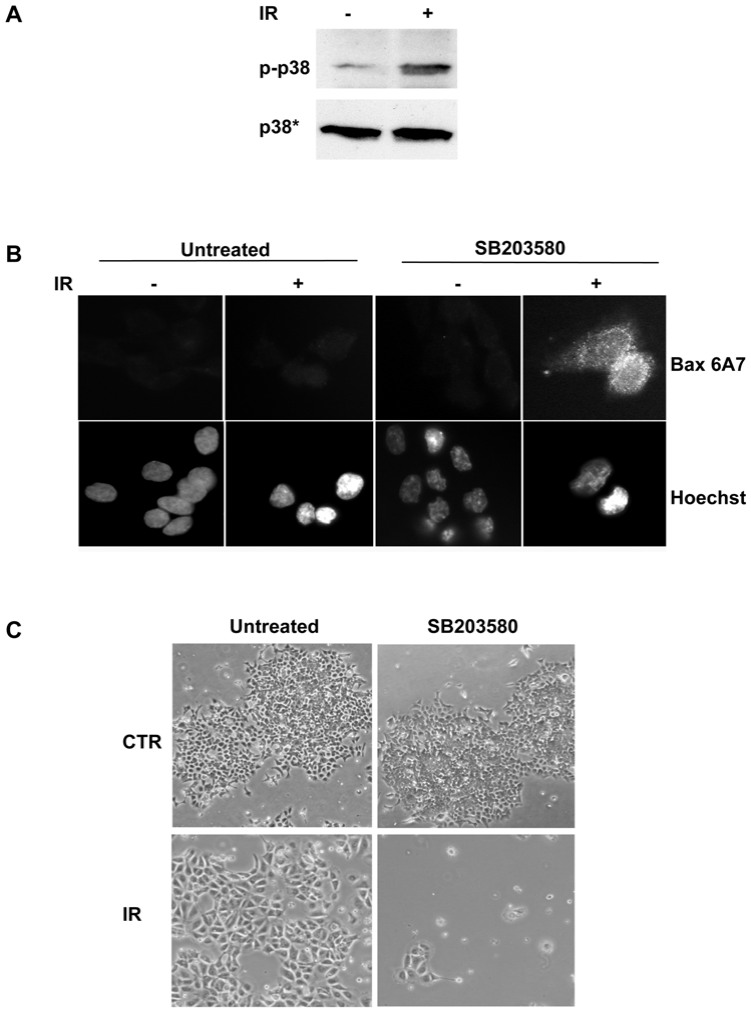
DNA damage activates p38 MAPK in HCT116 cells. A) HCT116 cells were left untreated or irradiated (20 Gy) and further incubated in complete medium for 3h. Samples were subjected to western blot analysis as described in Figure 5A. B) HCT116 cells, pre-treated with the specific p38 MAPK inhibitor SB203580 (20 μM) in complete medium for 30 min, were left untreated or irradiated (20 Gy) and further incubated for 48h. Cell death was assessed by detection of activated Bax, using the anti-Bax 6A7 antibody (upper panel). Nuclei were counterstained with Hoechst 33258 (lower panel). C) HCT116 cells, pre-treated with the specific p38 MAPK inhibitor SB203580 (20 μM) in complete medium for 30 min, were irradiated (20 Gy) and further incubated for 48h. Cells were visualized by contrast phase microscopy.

DNA damage was shown to induce activation of p38 MAPK [17]. We therefore tested whether exposure of HeLa cells to CDT or IR stimulates p38 MAPK activity, and whether this is required for protection from cell death. Both CDT intoxication and irradiation induced activation of p38 MAPK within 3h after treatment and this effect was maintained for at least 24h, as assessed by western blot using a p38 MAPK phospho-specific antibody (p-p38) (Figure 5A). The kinetics of p38 MAPK phosphorylation is in line with the previously reported kinetics of RhoA activation, which peaks at 4h after exposure to CDT or IR [6]. To determine whether p38 MAPK activation is important for cell survival, the cells were treated with the p38 MAPK specific inhibitors SB203580 or SB202190 (20 μM) prior to irradiation or intoxication. Inhibition of p38 MAPK was associated with a 2- to 4-fold increase in the number of cells exhibiting chromatin condensation 48h after treatment (Figure 5B and data not shown). The increased rate of cell death was confirmed by monitoring activation and increased expression of Bax (Figures 5C and 5D). Activation of p38 MAPK in response to DNA damage was also observed in the colorectal carcinoma cell line HCT116 upon irradiation (Figure 6A). As in HeLa cells, pre-treatment of HCT116 cells with SB203580 prior to irradiation was associated with increased activation of Bax (Figure 6B). Quantification of the Bax positive cells in this set of experiments was hampered by the low number of cells that survived irradiation upon pre-treatment with SB203580 (Figure 6C). These results indicate that activation of p38 MAPK protects the cells from death induced by DNA damage.

**Figure 7 pone-0002254-g007:**
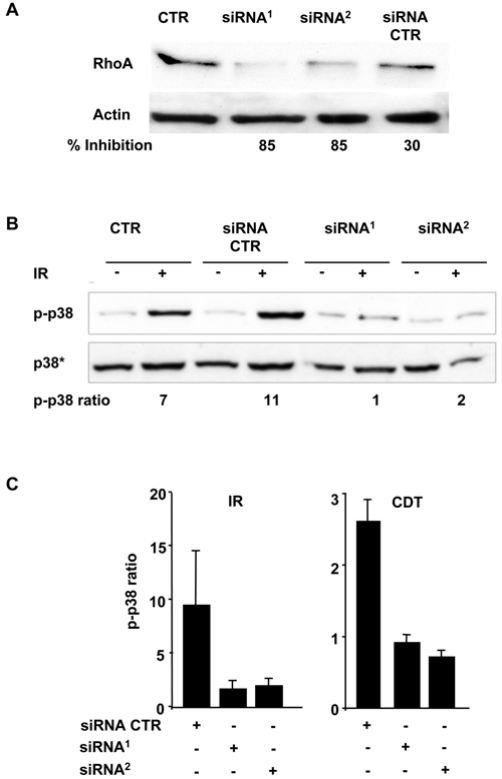
DNA damage-induced p38 phosphorylation is RhoA-dependent. A) HeLa cells were transfected with the control siRNA or two independent RhoA specific siRNA (siRNA^1^: Hs_RHOA_6; siRNA^2^: Hs_RHOA_7). Expression of the endogenous RhoA levels was analysed by western blot. Percentage inhibition was calculated as in Figure 2A. B) Untransfected HeLa cells or cells transfected with control siRNA, or two independent RhoA specific siRNA were left untreated or irradiated (20 Gy), and further incubated for 4h in complete medium. p38 phosphorylation was assessed as in Figure 5A. p-p38 ratio represents the ratio between the optical density of the phospho-p38 band in treated cells and optical density of the phospho-p38 band in the untreated cells. C) Quantification of the changes in the levels of p38 phosphorylation in HeLa cells transfected with control siRNA, RhoA specific siRNA^1^ or siRNA^2^. Mean±SD of 6 independent experiments performed with cells exposed to IR (left panel), and 3 independent experiments performed cells exposed to CDT (right panel) cells. p-p38 ratio is defined as in Figure 7B.

**Figure 8 pone-0002254-g008:**
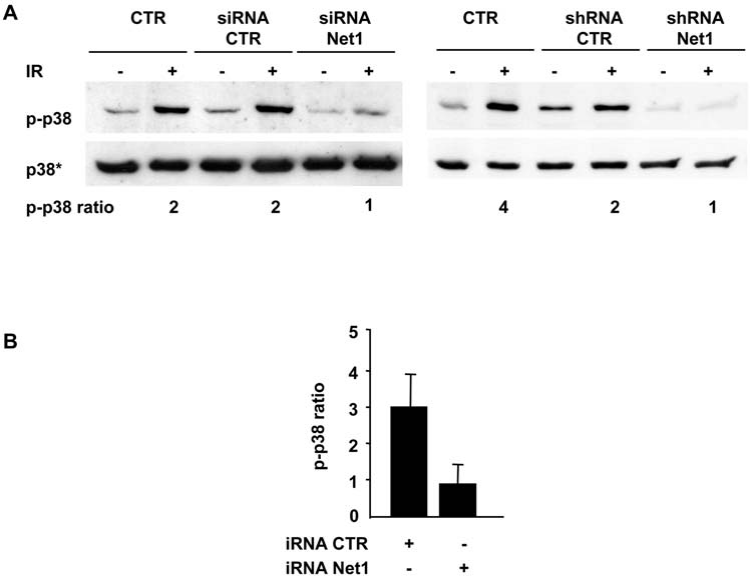
DNA damage-induced p38 phosphorylation is Net1-dependent. A) Untransfected HeLa cells or cells transfected with either the Net1 specific siRNA (left panel) or shRNA (right panel), or the relevant controls were left untreated or irradiated (20 Gy), and further incubated for 4h in complete medium. p38 phosphorylation was assessed as in Figure 5A. B) Quantification of the changes in the levels of p38 phosphorylation in irradiated HeLa cells transfected with control or Net1 specific iRNA. iRNA is defined as in Figure 2B. Mean±SD of 6 independent experiments (three performed with siRNA, and three performed with shRNA).

To assess whether the activation of p38 MAPK was dependent on RhoA and its regulator Net1, the levels of irradiation-induced phosphorylation were assessed in cells where the expression of the two proteins was independently knocked down by RNAi. Transfection with two independent RhoA specific siRNA oligonucleotides consistently induced a 70 to 80 percent reduction of the endogenous levels of RhoA (Figure 7A), and this effect was associated with a strong inhibition of p38 MAPK activation in response to IR and CDT (Figures 7B and 7C). Similar levels of inhibition were obtained by blocking RhoA activity using the cell permeable inhibitor C3 transferase (data not shown). Knock down of endogenous Net1 expression, by either siRNA or shRNA, resulted in an equally potent inhibition of p38 MAPK activation in irradiated cells (Figures 8A and 8B). These results indicate that Net1 and RhoA are upstream signals in the p38 MAPK activation cascade in response to DNA damage.

**Figure 9:**
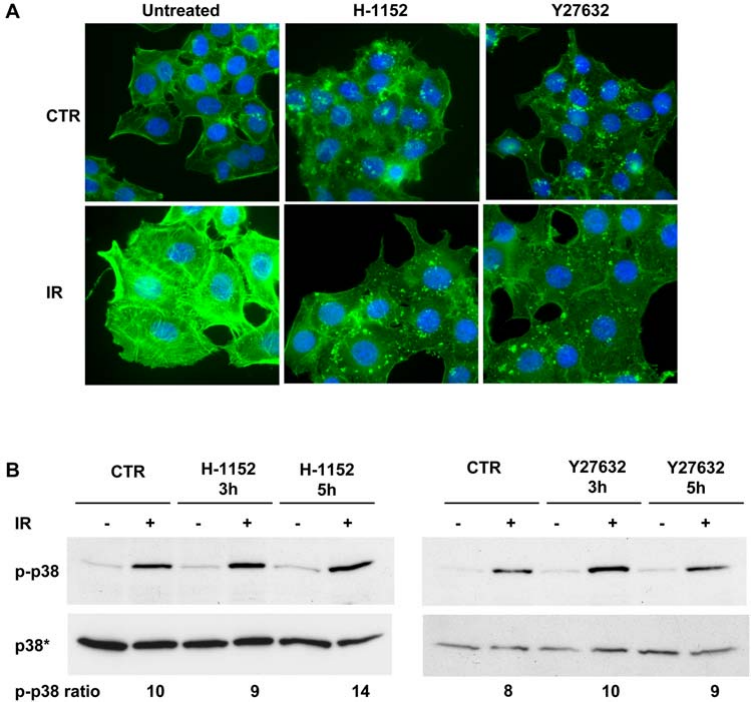
ROCK inhibitors do not prevent DNA damage-induced p38 MAPK phosphorylation. A) HeLa cells, were left untreated or pre-treated with the specific ROCK inhibitors H-1152 or Y27632 (10 μM) in complete medium for 1h prior to irradiation (20 Gy), and further incubated in the presence or absence of the inhibitor for 12h. The actin cytoskeleton was visualized by FITC-phalloidin staining (green). B) HeLa cells, pre-treated with the specific ROCK inhibitors H-1152 or Y27632 (10 μM) in complete medium for 1h, were left untreated or exposed to IR (20Gy), and further incubated in the presence or absence of the inhibitors for the indicated periods of time. p38 MAPK phosphorylation was assessed as in Figure 5A.

To determine whether the RhoA activated kinases ROCKI and ROCK II are required for p38 MAPK activation, HeLa cells were treated with the ROCKI/II inhibitors H-1152 or Y27632 prior to irradiation. As expected, pre-treatment of control HeLa cells with both inhibitors altered the organization of the actin cytoskeleton and prevented the formation of actin stress fibers upon irradiation (Figure 9A), confirming that these effectors were efficiently blocked. However, the treatment did not impair the activation of p38 MAPK (Figure 9B), indicating that ROCKI/II were not required for signalling to this kinase.

**Figure 10:**
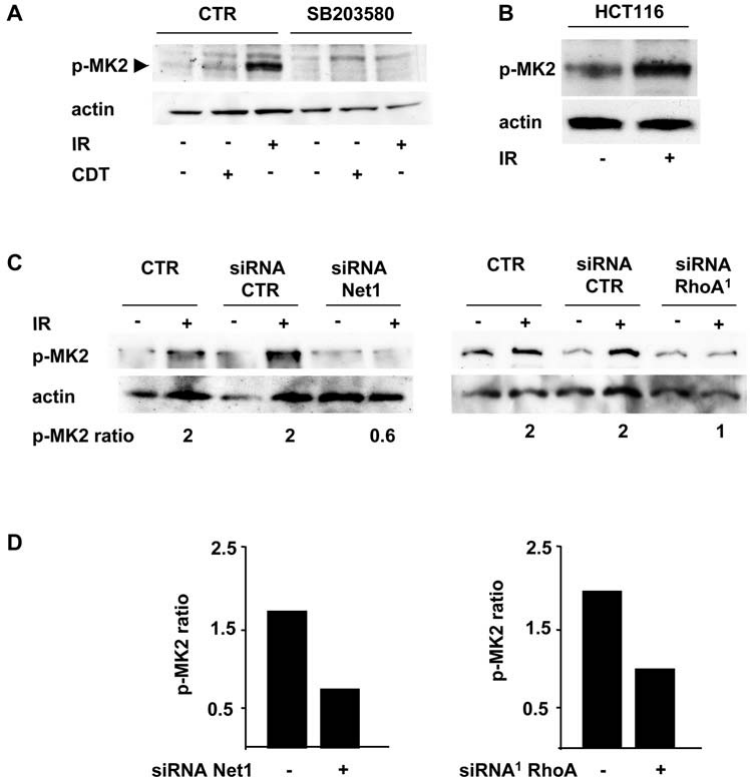
The p38 MAPK effector MK2 is phosphorylated in a Net1- and RhoA-dependent manner in response to genotoxic agents. A) Untreated HeLa cells, or cells pre-treated with the specific p38 MAPK inhibitor SB203580 (20 μM) in complete medium for 30 min, were exposed to CDT (2 μg ml^−1^) for 4h, or irradiated (20 Gy) and further incubated in complete medium for 4h. Samples were subjected to western blot analysis using antibodies specific for phospho-MK2 (p-MK2) or actin. B) HCT116 cells were left untreated or irradiated (20 Gy) and further incubated in complete medium for 3h. Samples were subjected to western blot analysis as described in Figure 10A. C) HeLa cells transfected with: i) control siRNA; ii) Net1 specific siRNA (left panel); iii) the RhoA specific siRNA^1^ (right panel) were left untreated or irradiated (20 Gy), and further incubated for 4h in complete medium. MK2 phosphorylation was assessed as in Figure 10A. p-MK2 ratio represents the ratio between the optical density of the phospho-MK2 band in irradiated cells and optical density of the phospho-MK2 band in the untreated cells. D) Quantification of the changes in the levels of MK2 phosphorylation in irradiated HeLa cells: mean of 3 independent experiments performed with the Net1 specific siRNA (left panel) and 2 independent experiment performed with the RhoA specific siRNA (right panel).

The MAPK-activated protein kinase 2 (MK2) is a direct substrate of the p38 MAPK α-and β-isoforms [18]. We asked therefore whether this protein is also activated in a Net1- and RhoA-dependent manner upon induction of DNA damage. As illustrated in Figure 10, a 2- to 4-fold increase in the phosphorylation of MK2 on its activating site Thr334 (p-MK2) was observed in HeLa cells 4h after irradiation or intoxication (Figure 10A), and a similar effect was observed in irradiated HCT116 cells (Figure 10B). As expected, this effect was prevented by pre-treatment with the p38 MAPK specific inhibitor SB203580 (Figure 10A). Importantly, the phosphorylation of MK2 following irradiation was abrogated by knock down of either Net1 or RhoA, indicating that these proteins are required for MK2 activation (Figures 10C and 10D).

## Discussion

The transforming ability of a truncated form of Net1 described by Chen et al. [12] suggests that this protein may be involved in regulating the delicate balance between cell growth and cell death. This possibility is substantiated by our finding that Net1 regulates the activation of RhoA and p38 MAPK, and promotes cell survival in response to genotoxic agents. Furthermore, our data identify genotoxic stress as a trigger for Net1 activation and contribute to the characterization of a novel DNA damage-induced survival pathway involving Net1 and its downstream targets RhoA and p38 MAPK.

### Net1 is activated and regulates RhoA-dependent actin stress fiber formation upon induction of DNA damage

Alberts *et al.* have previously shown that phosphorylation of Net1 on Ser152 prevents RhoA activation and concluded therefore that pSer152 inhibits the GEF activity of Net1 [15]. We now show that exposure to CDT or IR decreases the levels of pSer152 phosphorylation of the endogenous as well as an ectopically expressed Net1 (**Figure 1**), thus identifying genotoxic stress as a signal for Net1 activation in vivo. The mechanisms involved in Net1 de-phosphorylation remain unknown. A constitutively active form of the Rac1-activated protein kinase PAK1 (PAK1*) was identified as the Ser152-specific Net1 kinase *in vitro*, and expression of PAK1* prevented Net1-induced stress fiber formation in Swiss 3T3 cells [15]. We did not observe any significant change in the level of Rac1 or Cdc42 activation in HeLa cells or primary fibroblasts exposed to CDT or IR [6], and PAK1 activity was not changed within 30 min after irradiation, a time when the dephosphorylation of pS152-Net1 was maximal (data not shown). This suggests that the decrease in pSer152-Net1 observed in our experiments does not involve inactivation of PAK1. Conceivably, a different, as yet unknown Net1 specific kinase may be down-regulated. Alternatively, exposure to CDT or IR may enhance the rate of p-S152-Net1 de-phosphorylation by activation of a phosphatase.

Inhibition of endogenous Net1 by RNAi and expression of a dominant negative Net1 demonstrated that this GEF is required for RhoA activation and for the subsequent re-organization of the actin cytoskeleton in response to intoxication or irradiation (**Figures 2** **and** **3**). It is noteworthy that stress fiber formation is detected in epithelial cells that are arrested in G1 following treatment with TGF-β [19]–[21], and similar changes occur in cells exposed to bacterial toxins that inhibit, Cycle inhibiting factor (Cif) [22], or promote, *Pasteurella multocida* toxin (PMT) [23], cell cycle progression. Net1 is a major player in the morphological changes that characterize both TGF-β [21] and DNA damage (in this work). It is thereby tempting to speculate that Net1-regulated cytoskeleton rearrangements may be a common feature of the response to stress signals that deregulate the cell cycle.

### Net1/RhoA-dependent survival signals

Our RNAi experiments demonstrate that Net1 and RhoA are critical for protecting intoxicated and irradiated cells from cell death induced by DNA damage (**Figure 4**), and identify p38 MAPK as a key mediator in the delivery of the survival signals (**Figures 5** **to** [Fig pone-0002254-g006] [Fig pone-0002254-g007] [Fig pone-0002254-g008] **9**). The mechanism by which RhoA controls p38 MAPK phosphorylation remains still unclear. Marinissen at al. demonstrated that RhoA stimulates c-jun expression via activation of the p38γ MAPK isoform, resulting in aberrant cell growth and malignant transformation [24]. Interestingly, we have found that the activation of p38 MAPK in response to DNA damage was abrogated by SB203580 and SB202190 that are specific inhibitors for the α- and β-isoforms of p38 MAPK [25], suggesting that different isoforms may be targeted by RhoA depending on the triggering stimulus. A large number of effector proteins mediate signalling downstream of RhoA [26]. Since the RhoA activated kinases ROCKI and ROCKII regulate many of the cytoskeletal effects of RhoA, we examined whether they transduced the signal from RhoA to p38 MAPK. Interestingly, pre-treatment with pharmacological inhibitors of ROCK did not prevent p38 MAPK activation, but inhibited stress fiber formation (**Figure 9**), suggesting that RhoA may utilize different sets of effector proteins to control these cellular responses. This is likely to require the selective clustering of the downstream effectors on distinct scaffold proteins, as suggested by the finding that the RhoA-dependent activation of JNK involves the association of Net1 and the JNK activators MLK2, MLK3 and MKK7 with the scaffold protein CNK1, while this is not required for induction of stress fibers [27].

**Figure 11:**
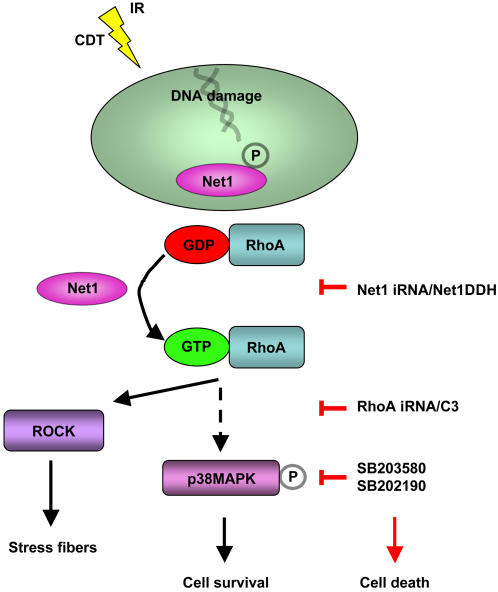
Summary of the Net1-regulated survival signals upon exposure to genotoxic agents. Upon intoxication or irradiation, Net1 is dephosphorylated and induces activation of RhoA, leading to a RhoA dependent phosphorylation of p38 MAPK and its downstream effector protein MK2. This signalling pathway can be blocked at different levels by: i) iRNA knock down of endogenous Net1 levels or expression of the dominant negative Net1ΔDH; ii) C3-mediated RhoA inhibition or iRNA knock down of endogenous RhoA levels; iii) p38 specific inhibitors. In each case, the effect of this interference results in enhanced cell death in response to genotoxic agents.

The current literature identifies JNK as the main MAPK induced by irradiation [17]. Consistent with our previous results showing that CDT or IR do not activate the JNK regulators Rac and Cdc42 [6], we detected only low levels of JNK phosphorylation and AP1 activation 4h and 12h after intoxication in HeLa cells (data not shown). In contrast, up to 10 fold increase of p38 MAPK phosphorylation was consistently observed both in HeLa and HCT116 cells (**Figures 5** **and** **6**). This result is not surprising since, depending on the experimental models, p38 MAPK was shown to contribute to either survival or death signals in response to DNA damage [17]. The downstream signals involved in the survival response remain unclear. Reinhardt at al. recently showed that the survival in p53-deficient fibroblasts exposed to cisplatin and doxorubicin is enhanced by ATM-dependent activation of p38 MAPK and its downstream effector MK2 [28]. This pathway has been defined as the third cell cycle-dependent checkpoint, in addition to the well-characterized ATM/Chk2 and ATR/Chk1 responses [28]. Our findings demonstrate that these signals operate also in tumor cells, such as HeLa and HCT116, since blockage of p38 MAPK activation by specific inhibitor abrogates their capacity to survive irradiation or intoxication (**Figures 5** **and** **6**). Furthermore, we have identified Net1 and RhoA as key molecules controlling the activation of this novel checkpoint pathway (**Figures 7** **and** **8**). A schematic illustration of the Net1-regulated signal cascade identified in this work is shown in **Figure 11**.

### Conclusions

Any event capable of promoting the survival of cell with damaged DNA is detrimental since it would favour tumor initiation and/or progression (reviewed in [29], [30]). Our finding that induction of DNA damage by CDT is accompanied by the concomitant activation of survival signals suggests that chronic infection with CDT producing bacteria may promote genomic instability and favour malignant transformation. The association between bacterial infections and cancer is poorly understood. The only bacterium classified as human carcinogen is *Helicobacter pylori*, but a possible involvement in oncogenesis has been suggested for other bacteria, such as for example the Gram-negative bacterium *Salmonella typhi* [31]. Indeed, several Gram-negative bacteria have been shown to produce DNA damaging toxins [32], [33]. The work described in this paper provides the first molecular characterization of a survival pathway triggered by CDT intoxication. Further dissection of this pathway will provide new tools to elucidate the mechanisms of bacterial-induced carcinogenesis, and may also help to design specific inhibitors that can act synergistically with conventional chemotherapy.

## Materials and Methods

### Cell lines and plasmids

HeLa and HCT116 cell lines were obtained from the ATCC and grown as described [6]. The plasmid expressing the c-Myc epitope-tagged Net1ΔDH was a kind gift from Drs. A. Hall and A. Schmidt (Medical Research Council Laboratory for Molecular Cell Biology, University College London, London, UK). The plasmid expressing the Net1A protein was previously described [16].

### CDT and treatments

Production of the *H. ducreyi* CdtA, CdtB and CdtC subunits and reconstitution of the active holotoxin (here named as CDT) was previously described [6], [34].

#### CDT intoxication

Cells were incubated for the indicated time periods with CDT (2 μg ml^−1^) in complete medium.

#### Ionizing radiation

Cells were irradiated (20 Gy), washed once with PBS and incubated for the indicated time periods in complete medium.

#### SB203580/SB202190 treatment

Cells were pre-treated with the p38 MAPK specific inhibitors SB203580 or SB202190 (20 μM) (Calbiochem) in complete medium for 30 min at 37°C, before exposure to CDT or IR.

#### ROCK inhibitors

Cells were pre-treated with the ROCK inhibitors H-1152 and Y27632 (10 μM) (Calbiochem) in complete medium for 1h at 37°C, before exposure to CDT or IR, and then further incubated in the presence of the inhibitor for the indicated periods of time.

### Immunofluorescence

#### Phalloidin staining

Phalloidin staining of actin filaments was performed as previously described [6].

#### Bax 6A7 staining

Cells were fixed with 4% paraformaldehyde, and permeabilized with 0.2% Triton X-100 for 2 min at 22°C. Antibody non-specific binding was blocked with 3% BSA in PBS for 30 min at 22°C. Slides were further incubated for 1h at 22°C with the conformation-specific monoclonal antibody (6A7, BD Pharmingen), which recognizes the activated form of Bax. Slides were washed three times with PBS and then incubated with TRITC-conjugated rabbit anti-mouse antibody (DAKO; diluted 1:100 in PBS) for 30 min at 22°C. Nuclei were counterstained with Hoechst 33258 (Sigma; 0.5 μg ml^−1^).

### iRNA

#### Net1 shRNA

The following oligonucleotides were used for production of Net1 specific shRNAs:

shRNA-A (target nucleotides: 312–332):

5′-TCTCAATCTCTCCTGTAAGAAATGGACACCATTTCTTACAGGAGAGATTCT-3′
5′-CTGCAGAATCTCTCCTGTAAGAAATGGTCTCCATTTCTTACAGGAGAGATT-3′

shRNA-B (targets nucleotides: 615–635):

5′-TCTCAAAGTTGTCCATCATGTCAGAACATCTGACATGATGGACAACTTCT-3′
5′-CTGCAGAAAGTTGTCCATCATGTCAGATGTTCTGACATGATGGACAACTTT-3′

shRNA-C (targets nucleotides: 819–837):

5′-TCTCCAAAGCTCTTCTTGATCAATTCAAGAGATTGATCAAGAAGAGCTTTGCT-3′
5′-CTGCAGCAAGCTCTTCTTGATCAATCTCTTGAATTGATCGSTCAAGAAGAGCTTTG-3′

Non targeting shRNA (scrambled sequence from Dlg1):

5′-TCTCGAGAATGCGAGGTCAAGTTCTTCCTGTCAAACTTGACCTCGCATTCTCCT-3′
5′-CTGCAGGAGAATGCGAGGTCAAGTTTGACAGGAACAACTTGACCTCGCATTCTC-3′

The oligonucleotides were annealed and ligated into the pGENECLIP-puromycin vector (Promega) according to the manufacturer's instructions.

Efficient down-regulation of the endogenous Net1 levels required co-transfection with the three Net1 specific shRNA expressing plasmids.

#### siRNA

The Net1 specific siRNA (HP validated 1027400), two RhoA specific siRNAs (Hs_RHOA_6 HP validated SI02654211, and Hs_RHOA_7 HP validated SI02654267), and the Alexa Fluor 488-labelled control siRNA (AATTCTCCGAACGTGTCACGT, 1022076) were purchased from Qiagen.

### Transfection

#### HA-Net1A

Two hundred thousand and one hundred thousand HeLa cells per well were grown in 12-well plates, or 24-well plates, respectively in complete medium. Transfections were performed with the indicated amount of the relevant plasmid using the Lipofectamine 2000 Reagent (Life Technologies), according to the manufacturer's instructions. Twenty-four hours after transfection, cells were either intoxicated or irradiated and incubated for the indicated time periods.

#### shRNA

One million Hela cells were grown in 6 cm diameter Petri dishes in complete medium, and transfected with the indicated shRNA plasmids (2 μg ml^−1^) using the Lipofectamine 2000 Reagent. Twenty-four hours after transfection, complete medium supplemented with puromycin (10 μg ml^−1^) was added and cells were further incubated for 72h.

#### siRNA

One hundred thousand HeLa cells were grown in 12-well plate in complete medium. Transfection was performed with 75 ng/well of the indicated siRNA with HiPerFect Reagent (Qiagen), according to the manufacturer's instructions, and cells were further incubated for 72h.

### RhoA activation

RhoA activation was assessed by the G-LISA™ RhoA Activation Assay Biochem Kit™ (Cytoskeleton) according to the manufacturer's instructions.

### Immunoprecipitation

Immunoprecipitations were performed as previously described [15]. The endogenous Net1 protein was immunoprecipitated using a goat α-Net1 specific antibody (Abcam).

### Western blot analysis

The following antibodies were used: α-HA, α-Bax (B-9) (Santa Cruz Biotechnology), α-phospho-Net1-Ser152 [15], α-Net1 specific rabbit serum, α-Net1 goat serum (Abcam), α-actin (Sigma), α-phospho-p38, α-p38, α-phospho-MK2 (Thr334), α-RhoA (Cell Signaling), and α-PARP (BD Biosciences). Blots were developed with enhanced chemiluminescence, using the appropriate horseradish peroxidase-labelled secondary antibody, according to the instructions of the manufacturer (GE Healthcare).
